# Phylogenetic Relationships among Deep-Sea and Chemosynthetic Sea Anemones: Actinoscyphiidae and Actinostolidae (Actiniaria: Mesomyaria)

**DOI:** 10.1371/journal.pone.0010958

**Published:** 2010-06-04

**Authors:** Estefanía Rodríguez, Marymegan Daly

**Affiliations:** 1 Division of Invertebrate Zoology, American Museum of Natural History, New York, New York, United States of America; 2 Department of Evolution, Ecology, and Organismal Biology, The Ohio State University, Columbus, Ohio, United States of America; University of Canterbury, New Zealand

## Abstract

Sea anemones (Cnidaria, Actiniaria) are present in all marine ecosystems, including chemosynthetic environments. The high level of endemicity of sea anemones in chemosynthetic environments and the taxonomic confusion in many of the groups to which these animals belong makes their systematic relationships obscure. We use five molecular markers to explore the phylogenetic relationships of the superfamily Mesomyaria, which includes most of the species that live in chemosynthetic, deep-sea, and polar sea habitats and to test the monophyly of the recently defined clades Actinostolina and Chemosynthina. We found that sea anemones of chemosynthetic environments derive from at least two different lineages: one lineage including acontiate deep-sea taxa and the other primarily encompassing shallow-water taxa.

## Introduction

Since their discovery thirty years ago, biological communities at chemosynthetic sites such as hydrothermal vents, cold seeps, and whale falls, have proven to be a rich source of new species, attracting the interest of scientists [1]–[4]. Many of the species found in these habitats have morphologies or biologies distinct from those of their close relatives. The organisms are inferred to have adapted to the distinctive chemical, temperature, and biological circumstances of their particular chemosynthetic habitats (reviewed in [5] [Bibr pone.0010958-Childress1]–[7]). The physical conditions of chemosynthetic habitats are presumed to exclude organisms that lack mechanisms for coping with high temperatures, sulfide, methane, or metal concentrations [6].

The occurrence of unique organisms and the absence of members of the “background” fauna contribute to the remarkable degree of endemism reported for chemosynthetic environments [8]. Even more remarkable is the high endemism at a supraspecific taxonomic level (family or genus) suggesting ancient origins of the fauna (back to the Paleozoic, 540–248 ma), with a long and continuing evolutionary history [9]. Because of their reliance on an *in situ* chemosynthetic food source, these communities may have had an independent evolutionary history [2], [10]. Chemosynthetic environments also have been posited as stable refugia from global extinction events that devastated biological diversity in euphotic zones [2], [10], [11]. However, other interpretations of the diversity of chemosynthetic habitats postulate relatively recent (late Cenozoic) *in situ* radiations, with taxa from shallow water repopulating these environments after global extinctions events in the late Cretaceous and Cenozoic [12], [13]. Since different taxa show different evidence of evolutionary history (reviewed in [14] [Bibr pone.0010958-Yamaguchi1]–[16]), robust patterns are not established yet [13]. More comprehensive phylogenetic studies are required to elucidate the origin and history of this endemic chemosynthetic fauna.

Sea anemones (Phylum Cnidaria, Order Actiniaria) are present in all marine ecosystems, including chemosynthetic environments [17]. Some of these actiniarians inhabit the vent zone while others can be considered peripheral fauna associated with vents and seeps. Although present, sea anemones are not very abundant in the Atlantic and Pacific vent systems, but they dominate some microhabitats on the East-Lau Spreading Center (ELSC) in the Indian Ocean [18].

The high level of endemicity of chemosynthetic sea anemones and taxonomic confusion in many of the groups to which these animals belong makes their systematic relationships obscure [18], [19]. Prior to the description of *Boloceroides daphneae* [20], a species from the vent periphery of the East Pacific Rise (EPR), all taxa found at chemosynthetic habitats were described within Actinostolidae, a family mainly comprised of polar and deep-sea taxa [21], [22]. Traditionally, actinostolids have been placed in Mesomyaria, one of the two major superfamilies of Actiniaria characterized as having a mesogleal marginal sphincter [21], [22]. Molecular phylogenetic analyses support the grouping of members of Mesomyaria together with the other lineage having mesogleal marginal sphincter muscles and with a few lineages whose members lack marginal musculature, in a group called the Acontiaria-Boloceroidaria-Mesomyaria clade (ABM, see [23], [24]). The ABM clade includes acontiate actiniarians, a lineage characterized by acontia (nematocyst-dense, thread-like extensions of the mesenterial filaments); boloceroidarians, a small group of sea anemones with longitudinal musculature in the column; and mesomyarians.

Recent descriptions of new taxa and morphological phylogenetic analysis led to a rearrangement of the taxonomy of Actinostolidae to better fit these new findings. The genera of Actinostolidae previously reported from chemosynthetic environments were transferred to Actinoscyphiidae [19]. Our morphological analysis [19] suggested that polar taxa within Actinostolidae form a clade (called Actinostolina), and that within Actinoscyphiidae is Chemosynthina, a clade containing the genera reported from hydrothermal vents and cold seeps. In some of the equally parsimonious topologies, the whale-fall endemic species *Anthosactis pearseae* is the sister group to Chemosynthina. These phylogenetic trees raise the possibility of a single lineage of taxa inhabiting chemosynthetic environments within Actiniaria, a highly derived lineage that lost acontia [19]. Furthermore, the relationship between actiniarians from chemosynthetic habitats and *Actinoscyphia* suggests that sea anemones of chemosynthetic environments derived from ancestors living in deep-sea. However, new discoveries confound simple explanations for the diversity and history of these environments: a new (undescribed) species from EPR contradicts the hypothesis of a single lineage for sea anemones from chemosynthetic environments.

We explore the phylogenetic relationships of sea anemones in the superfamily Mesomyaria using five molecular markers (12S, 16S, 18S, 28S and COIII). The majority of these species are from deep-sea, chemosynthetic, and polar environments. We test the monophyly of the recently defined Actinostolina and Chemosynthina [19]. We find that sea anemones of chemosynthetic environments are the product of at least two radiations: one from adjacent deep-sea fauna and the other probably from shallow water.

## Materials and Methods

### Taxonomic sampling and data collection

We provide new sequences for multiple representatives of each family within the ABM clade (Table 1). Multiple species were sampled for potentially related sister groups or taxa with ambiguous placement in previous analysis such as Edwardsiidae (see [23]). We include multiple representatives of Endomyaria, the sister clade of ABM. These genera span the diversity of Actiniaria and thus provide a strong test of monophyly of mesomyarian sea anemones. We root our analysis with *Savalia savaglia*, a member of the order Zoanthidea, the sister group of Actiniaria [25]. We have included only those taxa from which we were able to amplify at least three of the five markers, and thus have analyzed a total of 301 sequences for 63 taxa. Comparative sequences from GenBank were also included as appropriate (Table S1).

**Table 1:** Results of parsimony analysis of each data set.

Marker or data set	Marker aligned length	# Parsimony Informative Characters (% informative)	# Equally parsimonious trees	Tree Length	# taxa
COIII	714	236 (33.0%)	59	1192	59
12S	976	171 (17.5%)	21	679	55
16S	569	148 (26.0%)	90	627	63
18S	2154	292 (13.6%)	50	1630	63
28S	1203	452 (37.6%)	8	3180	61
Mitochondrial	2259	554 (24.5%)	12	2786	64
Nuclear	3357	744 (21.7%)	9	4919	64
Combined	5616	1298 (23.1%)	1	8145	64

Specimens were collected by hand intertidally, through SCUBA diving, via trawls or using Remote Operate Vehicles (ROV). All specimens were identified using polyp anatomy and the distribution and size of cnidae in various regions of the polyp. Voucher specimens in formalin have been deposited at the American Museum of Natural History (AMNH), the Bavarian State Collection of Zoology (ZSM), the collection of Biodiversidad y Ecología de Invertebrados Marinos (BEIM) at the University of Seville, California Academy of Sciences (CAS), Field Museum of Natural History (FMNH), University of Kansas Natural History Museum (KUNHM), and the U. S. National Museum of Natural History (USNM).

During this work, we discovered one specimen of a new species of sea anemone from the EPR. A preliminary identification of the specimen reveals that it is a new species: it has an endodermal marginal sphincter, a character unknown among sea anemones from chemosynthetic environments. The specimen has been preliminary identified as *Isotealia* sp. nov. It does not match any described species of *Isotealia* in anatomy or cnidom. We decided to include this specimen in our analysis despite the preliminary state of its identification because of its relevance to the aim of this work. The formal morphological description of this new taxon is currently in progress and will be published after a thorough comparison with morphologically related taxa, a goal beyond the aim of this contribution. Genomic DNA was isolated from tentacle or column tissue using the Quiagen DNAeasy® kit or standard CTAB extraction. We sequenced three mitochondrial (partial 12S and 16S rDNA and COIII) and two nuclear (18S and partial 28S rDNA) markers from genomic DNA using primers published previously (12S: [26]; 16S, COIII: [27]; 18S: [28]; 28S: [29]). Samples which could not be readily amplified using standard protocols were amplified with the high-fidelity enzyme Herculase® (Stratagene, La Jolla, CA), using manufacturer supplied protocols. All PCR products were cleaned using AmPure® magnetic bead solution (AgenCourt, Beverly, MA) and rehydrated with deionized, double-distilled water. Sequencing reactions used a total of 10 µL of cleaned PCR product, at a concentration of 25 ng product for every 200 base pairs of marker length. Cleaned PCR products were sequenced using amplification primers on an ABI 3730*xl* by staff at the sequencing facilities of Genaissance (New Haven, CT) and Cogenics (Houston, TX). Forward and reverse sequences were assembled in Sequencher v4.8 (Gene Codes Corporation, Ann Arbor, MI) and blasted against the nucleotide database of GenBank to determine whether the target locus and organism were sequenced rather than a symbiont or other contaminant. All sequences have been deposited in GenBank (Table S1).

### Data analysis

Sequences for each marker were aligned in Muscle [30] using the default parameters. The Incongruence Length Difference test (ILD: [31], [32]) was used to identify instances of incongruence within and between the nuclear and mitochondrial markers. The resulting alignments and the combined data sets of mitochondrial, nuclear, and all markers were analyzed using random and consensus sectorial searches, tree drifting, and 100 rounds of tree fusing in TNT v1.1 [33]. In all analyses, gaps were treated as ambiguous (?) rather than as a fifth state. Trees of minimum length were found at least three times. The combined data were subjected to 1000 rounds of jackknife resampling (36% probability of removal, collapse clades with <50% support) to assess support for clades.

The appropriate model of nucleotide substitution for each gene was evaluated using Modeltest 3.7 [34]; selection of models was based on the Akaike Information Criterion (AIC), which rewards models for good fit but penalizes them for unnecessary parameters [35]. This combined alignment is archived in TreeBase (http://www.treebase.org/treebase/index.html), and was used for all phylogenetic inference. Maximum likelihood analyses were performed in RAxML 7.0.4 [36], using 1000 replications. Model parameters were estimated by RAxML. Clade support was assessed with 1000 rounds of bootstrap re-sampling.

## Results and Discussion

### Parsimony analysis

The markers range in length from 569 to 2154 bases, and contained 148–452 parsimony informative sites after alignment (Table 1). Trends in marker variability followed those previously reported for actiniarians: the longest marker (18S) was the least variable and nuclear markers were slightly less variable than mitochondrial ones [23], [24].

In addition to independent analyses of each marker, a mitochondrial (12S+16S+COIII) and a nuclear (18S+28S) data set, we combined all five markers into a simultaneous analysis of mitochondrial and nuclear sequences. Based on previous analyses of actiniarian DNA sequences (e.g., [23] [Bibr pone.0010958-Gusmo1]–[25]) and studies in other taxa [28], [37], we expect the most robust and well-supported tree to result from such an analysis. There are no known processes that would lead to discordant phylogenies for the mitochondrion and nuclear genomes in actiniarians. The ILD test did not detect significant incongruence for the comparison between mitochondrial and nuclear data sets nor within mitochondrial (12S, 16S and COIII) and nuclear (18S and 28S) data sets at p = 0.05.

**Figure 1:**
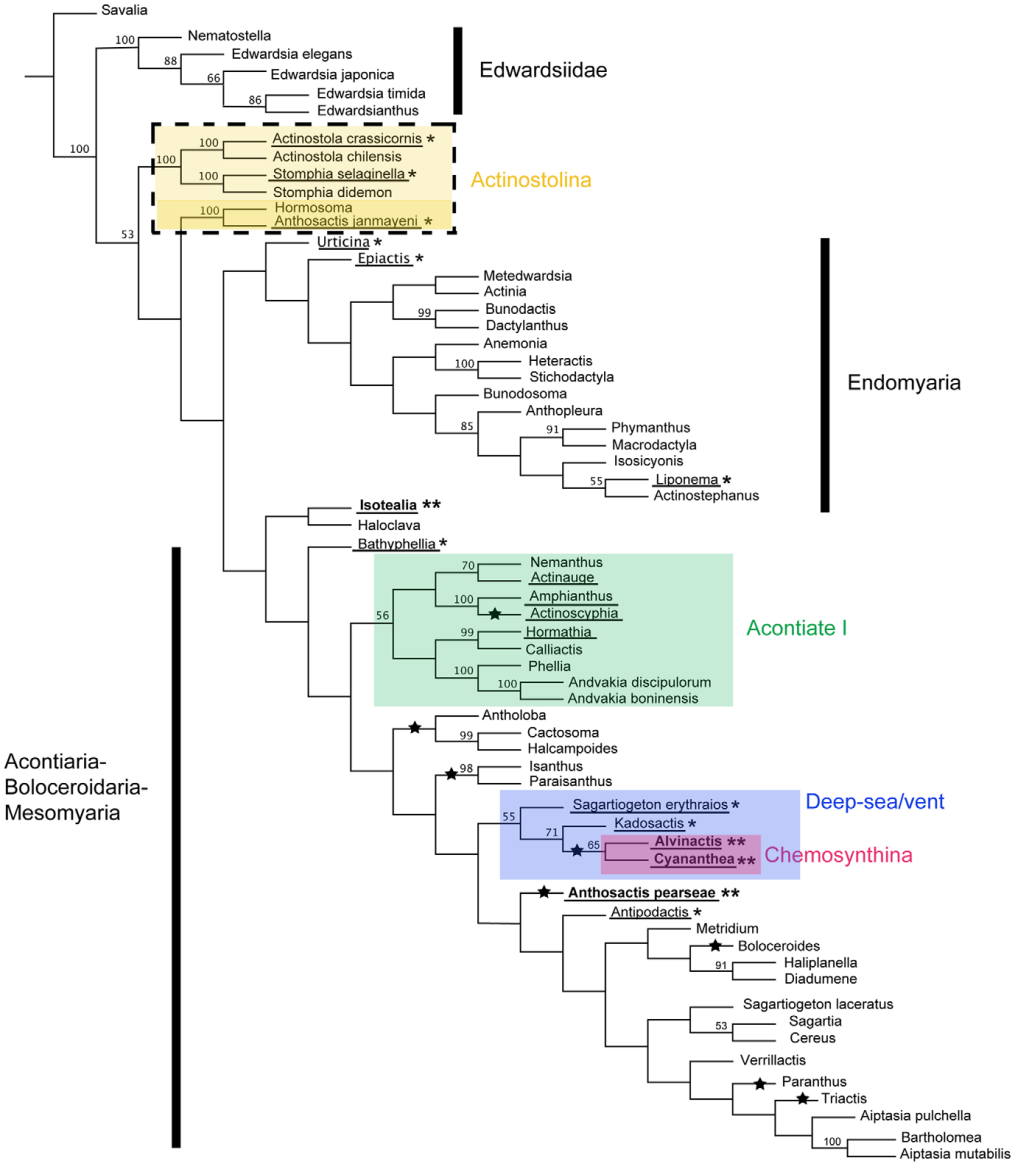
Parsimony tree. Tree resulting from combined parsimony analysis of COIII, 12S, 16S, 18S, and 28S data sets. Species epithets are given only for genera represented by more than one species; for complete list of taxa, see Table S1. Numbers above the branches are jackknife resampling values expressed as a percent; numbers <50 not indicated. Lineages marked with a star are inferred to have lost acontia. Taxa from chemosynthetic environments in bold and followed by two asterisks; those from deep seas (>1000 m) underlined and followed by one asterisk. Shaded boxes indicate clades defined in the text; name of each clade is next to the shaded box.

**Figure 2:**
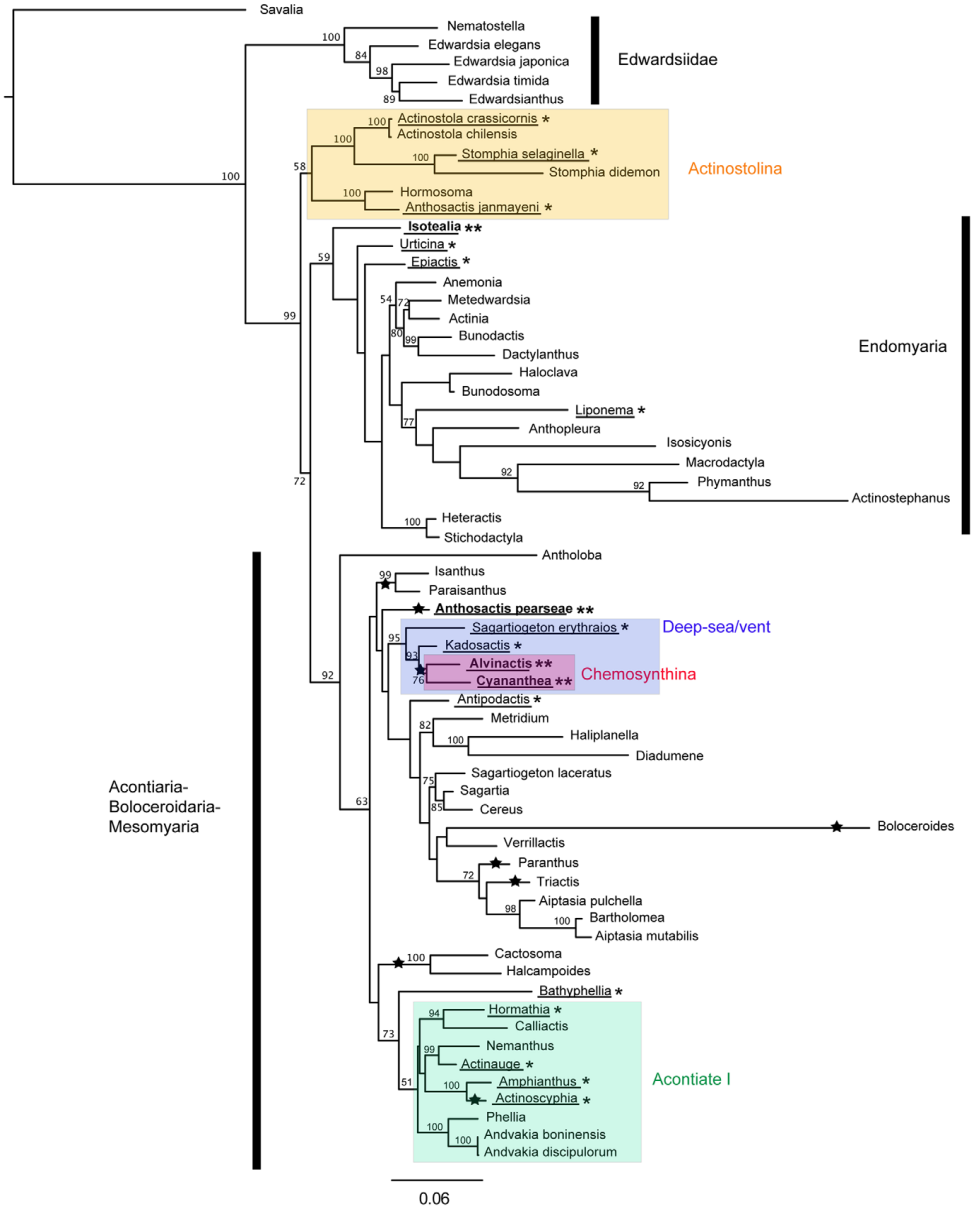
Maximum likelihood tree. Maximum likelihood tree resulting from combined analysis of COIII, 12S, 16S, 18S, and 28S data sets under the model GTR+ gamma. Relative branch lengths indicated. Species epithets are given only for genera represented by more than one species; for complete list of taxa, see Table S1. Numbers above the branches are bootstrap resampling values expressed as a percent; numbers <50 not indicated. Lineages marked with a star are inferred to have lost acontia. Taxa from chemosynthetic environments in bold and followed by two asterisks; those from deep seas (>1000 m) underlined and followed by one asterisk. Shaded boxes indicate clades defined in the text; name of each clade is next to the shaded box.

Each data set showed resolution at different levels in consensus, and depicted slightly different relationships; however, disagreements seem to be confined to basal nodes. Many of these differences can be explained by divergent sequences for specific markers for some taxa and the resulting “long-branch” attraction of these taxa [38], [39]. An example is the relationship between *Isotealia*-*Haloclava* in the parsimony tree (Figure 1): the long branches of both taxa are distinct in the likelihood analysis (Figure 2), and previous analyses [23], [25], [40] including those with broader sampling of Endomyaria, find *Haloclava* nested within Endomyaria. Thus, the relationship between *Isotealia* and *Haloclava*, and the placement of this clade at the base of the ABM clade is suspect. For this and other suspect placements (e.g., *Liponema* + *Actinostephanus*, *Boloceroides* among Diadumenidae), not all sequences were available for every taxon (Table S1). Because the effect of these artifacts is buffered in the combined data set [37], we show and discuss only those results.

The basic topology of the tree derived from parsimony analysis of the combined data set shows a monophyletic Edwardsiidae basal to the rest of the nynanthean actiniarians; this relationship is well supported with 100% jackknife frequency (see Figure 1). Note that *Metedwardsia* is recovered among Endomyaria rather than Edwardsiidae (Figure 1); this placement is concordant with the morphology of this genus (e.g., [41], Daly pers. obs.) although it disagrees with current taxonomy. Mesomyaria is polyphyletic, with members distributed at least in three clades. One clade includes Actinostolina members *Actinostola* and *Stomphia*, these are basal to a second mesomyarian clade that includes *Hormosoma* and *Anthosactis janmayeni*, other members of Actinostolina. This second mesomyarian clade is basal to a large clade that includes two previously resolved clades: Endomyaria and ABM (see [23]). However, there is no support for the node dividing Actinostolina into a paraphyletic grade. The remaining mesomyarian taxa in our analysis are distributed across the ABM clade, including the chemosynthetic taxa with a mesogleal marginal sphincter (*Alvinactis* and *Cyananthea*) and *Antholoba*, the remaining included member of Actinostolina. All actiniarians with an endodermal marginal sphincter except the chemosynthetic *Isotealia* are recovered within the Endomyaria clade. This chemosynthetic endomyarian is recovered as sister of *Haloclava* in a clade basal to ABM but without support. ABM contains several subclades, but only two of them are relatively well supported in our analysis. The first of these is a deep-sea/vent clade that includes the mesomyarian taxa *Alvinactis* and *Cyananthea* as sister taxa, clustering then with the deep-sea acontiates *Kadosactis* and *Sagartiogeton erythraios*. A close relationship between *S. erythraios* and *Kadosactis* has been proposed based on morphology [18]. All members of this clade are known only from depths exceeding 1000 m or from chemosynthetic environments. The second is the clade that includes *Phellia* + *Andvakia* and Hormathidae + *Nemanthus* + *Actinoscyphia* (named Acontiate 1 hereafter, 56% jackknife). This clade renders families Sagartiidae and Hormathiidae polyphyletic, concordant with previous results [23], [24]. *Anthosactis pearseae*, known only from whale-falls, does not nest with the deep-sea/vent clade or with *Actinoscyphia* as suggested by morphological analysis [19]. Within the ABM clade, some terminal relationships are recovered consistently (e.g., monophyletic family Isanthidae, a monophyletic Aiptasiidae sister to the aliciid *Triactis*, a sister relationship between *Haliplanella* and *Diadumene*), but disagreements in the basal nodes render deeper relationships within ABM unclear. The sister relationship between *Diadumene* and *Haliplanella* substantiates Manuel's [42] synonymy of these taxa, but does not address the issue of family-level synonymies, as these analyses do not include *Tricnidactis*, the lone genus remaining in Haliplanellidae (see [43]).

Parsimony analysis supports the previously recovered relationship between Boloceroidaria and the acontiate taxa but not the sister relationship between *Boloceroides* and *Triactis* (see [23]). *Boloceroides* is resolved as the sister of *Haliplanella-Diadumene* instead, a relationship that is difficult to explain in terms of morphology. However, *Boloceroides* has very divergent sequences, with a distinct long-branch in the likelihood analysis (Figure 2), and therefore this relationship is suspect [38], [39].

### Model-based analyses

The basic topology of the tree of highest likelihood coincides roughly with the parsimony (see Figures 1, 2); the two major differences correspond to nodes with low support in the parsimony tree. The tree of maximum likelihood contains a basal, well-supported, monophyletic Edwardsiidae. The Endomyaria and ABM clades are also recovered; however, the Endomyaria clade of the likelihood tree includes *Isotealia* and *Haloclava* (not as sister taxa) with moderate support (59% bootstrap). In the likelihood tree, *Haloclava* is the sister of *Bunodosoma*, a relationship previously recovered [23], [25], [40] but without support. As in the parsimony tree, Mesomyaria is polyphyletic, but the mesomyarian taxa are spread between two clades rather than three: i) one corresponding to Actinostolina with the exclusion of *Antholoba* (see [19]), which is basal to Endomyaria + ABM; and ii) within ABM.

The two well-supported subclades of ABM resolved in the parsimony tree are also resolved by the likelihood analysis. In this case, *Anthosactis pearseae* is basal to the deep-sea/vent clade but without support. Most terminal relationships recovered by parsimony are also recovered by likelihood (e.g., a monophyletic Aiptasiidae sister to the alicid *Triactis*, a sister relationship between families Haliplanellidae and Diadumenidae, monophyletic Isanthidae, etc.). In the likelihood analysis, *Boloceroides* groups with *Verrillactis* rather than with *Haliplanella* and *Diadumene*.

### Relationships among sea anemones from the deep-sea and chemosynthetic habitats

Despite the slight disagreement between the parsimony and the model-based analyses, both approaches show that sea anemones from chemosynthetic habitats derive from different lineages: i) one associated with Endomyaria, and ii) one or more within the ABM clade. The sole endomyarian from chemosynthetic habitats is *Isotealia* sp. nov., a recently discovered species known from a single specimen. The clade to which it belongs primarily encompasses shallow-water taxa, although at least four genera (*Epiactis*, *Isotealia*, *Liponema*, *Urticina*) have members that reportedly live in deep waters [21], [22]. Despite the relationship of *Isotealia* to other endomyarians is obscure, we are confidant it belongs within Endomyaria: all taxa with an endodermal marginal sphincter are consistently recovered together within the same clade in every study until now [23], [24], [25]. An endodermal marginal sphincter is one of the few morphological characters that seem to have phylogenetic information for sea anemones up to now.

Our analyses support previous hypothesis of loss of acontia within taxa living in chemosynthetic environments [19], [44]. However, because we find the deep-sea/vent clade only distantly related to *Actinoscyphia*, the particular relationships specified in that hypothesis are not supported, nor is the recent placement of *Alvinactis* and *Cyananthea* within Actinoscyphiidae (see [19]). *Actinoscyphia* is the sister taxa to *Amphianthus* in both analyses (within Acontiate 1 clade), with very high support; this relationship is also recovered by Gusmão and Daly [24]. Those species of sea anemone living in deep-sea chemosynthetic habitats are inferred to have lost acontia independently of deep-sea species like *Actinoscyphia*.

*Cyananthea* and *Alvinactis*, taxa within the proposed Chemosynthina clade, are recovered as sisters in all analyses. However, because we were able to sample DNA from only two of the six proposed genera in Chemosynthina, the validity of Chemosynthina is not clear. In any case, the original diagnosis of this clade has to be modified to specify that it includes only taxa from chemosynthetic habitats with a mesogleal marginal sphincter. This will make clear the exclusion of *Isotealia* n. sp., the recently discovered species from the EPR.

In this analysis, the closest relative of Chemosynthina is *Kadosactis*, a deep-sea and polar specialist [45], [46]. The relationship of these taxa to the two samples from Chemosynthina suggests that *Alvinatis* and *Cyananthea* (and possibly other members of Chemosynthina) belong in Kadosactidae, a homogeneous monogeneric family [46] until the recent inclusion of the vent genus *Seepactis* [44]. However, as currently defined, Kadosactidae contains only acontiate sea anemones [44], [45], and thus *Alvinatis* and *Cyananthea* cannot easily be included within that group. Acontia, while clearly labile phylogenetically, are significant in terms of identification, and thus generally have been accorded great significance in taxonomy. Furthermore, as relationships within ABM remain especially unclear at the level of family and genus, and as not all relevant taxa have been studied, we refrain from making taxonomic changes at this point.

The whale-fall endemic species *Anthosactis pearseae* does not group with *Anthosactis janmayeni,* its polar congener and type of the genus. No analysis or data set supports an exclusive relationship between these species, which are consistently recovered in different major clades. The lack of relationship between the two putative species of *Anthosactis* and the equivocality of the original generic assignment [47] argues for a new generic assignment for *A. pearseae*, perhaps as part of a much-needed revision of *Anthosactis* [19].

We recover no evidence in support of a close relationship between deep-sea and polar species, as has been found for other organisms (e.g., Scleractinia: [48]; nematodes: [49]). The polar-inhabiting Mesomyaria largely form a group ( =  Actinostolina, excluding *Antholoba*). The deep-sea taxa (including those from chemosynthetic habitats) are more broadly distributed in the trees, belonging to several clades (Figures 1, 2). With the exception of the deep-sea/vent clade, the deep-sea species are not part of habitat-specific clades. However, because this was not the focus of our analysis, our sampling strategy is not optimal for this question, and the robustness of this pattern needs to be examined with more concentrated sampling in those lineages.

### Summary and conclusions

Our phylogenetic analyses of molecular data from a broad sample of sea anemones, including species from deep-sea, polar, and chemosynthetic environments support at least two origins for those species inhabiting chemosynthetic habitats. One of these is probably within Endomyaria, a group not previously known to include any species endemic to chemosynthetic environments (reviewed in [18]). The phylogenetic placement of the endomyarian *Isotealia* sp. nov. is ambiguous, as it differs between the parsimony and likelihood analyses and is never well-supported. In contrast, the placement of the clade previously called Chemosynthina within the ABM clade is well-supported in all analyses. None of the molecular analyses support the inclusion of the whale-fall endemic *Anthosactis pearseae* in this clade, although *A. pearseae* is frequently associated with the larger clade that includes Chemosynthina.

Taxonomic placement of the sea anemones endemic to chemosynthetic habitats based on morphology is problematic. The two species belonging to Chemosynthina included in this analysis are not interpreted as closely related to *Actinoscyphia*, contradicting the relationship supposed by their inclusion in family Actinoscyphiidae by Rodriguez et al. [19]. Instead, these species are more closely related to the acontiate *Kadosactis.* Similarly, *Anthosactis pearseae* is not closely related to its congener *A. janmayeni*, casting doubt on its generic (and familial) placement. Nonetheless, the morphological distinction between clades Actinostolina and Chemosynthina is justified, as these groups are individually coherent but widely dispersed in the tree.

Our analyses find multiple lineages of mesomyarians (in the sense of actiniarians with a mesogleal marginal sphincter and without acontia): those that never had acontia (corresponding with Actinostolina to the exclusion of *Antholoba*) and those that lost acontia (mixed among ABM clade). This broadly agrees with the split implied by Carlgren's (1949) monograph between the family Actinostolidae (major component of the superfamily Mesomyaria) and the acontiate nynantheans (as a rule with a mesogleal sphincter too). However, Carlgren did not take into account the loss of acontia in several lineages (e.g., *Actinoscyphia,* Isanthidae), including these with Actinostolidae in Mesomyaria.

The sea anemones endemic to chemosynthetic habitats are not particularly closely related to those from polar seas. The polar taxa belonging to Actinostolina cluster together outside of the split between Endomyaria and the ABM clade; the deep and polar *Antipodactis* lies within ABM. Neither is closely allied to *Isotealia* sp. nov., *Anthosactis pearseae*, or Chemosynthina. Of the included species from chemosynthetic habitats, only those belonging to Chemosynthina are resolved with support in a consistent manner, and these are always found within a clade of acontiate deep-sea species. The position of *Isotealia* sp. nov. within Endomyaria remains enigmatic, perhaps because close relatives have not been included. Nevertheless, actiniarians with an endodermal marginal sphincter have consistently been recovered within Endomyaria, and this feature appears to be one of the few historically important taxonomic features with phylogenetic significance [23]. Because of the consistency of this feature, and the nature of the ambiguity of the placement of *Isotealia* sp. nov., we consider a close relationship between *Isotealia* sp. nov. and Endomyaria highly probable.

## Supporting Information

Table S1 Taxa included in this study, with voucher location and accession numbers.
(0.16 MB DOCX)
