# Supplementary material for: Phylogenetic Relationships among Deep-Sea and Chemosynthetic Sea Anemones: Actinoscyphiidae and Actinostolidae (Actiniaria: Mesomyaria)
Source: PLoS One. 2010 Jun 4;5(6):e10958. doi: 10.1371/journal.pone.0010958 (PMC2881040; doi:10.1371/journal.pone.0010958)
Supplement: Table S1 — Taxa included in this study, with voucher location and accession numbers. (0.16 MB DOCX) [file pone.0010958.s001.docx]

**Table S1.** **Taxa included in this study, with voucher location and accession numbers.**

| **Higher Taxon** | **Family** | **Genus** | **Species** | **Voucher** | **12S** | **16S** | **18S** | **28S** | **COIII** |
| --- | --- | --- | --- | --- | --- | --- | --- | --- | --- |
|  | Andvakiidae | ***Andvakia*** | *boninensis* | KUNHM | EU190717 | EU190759 | EU190848 | EU190805 | FJ489479 |
|  |  | ***Andvakia*** | *discipulorum* | KUNHM | GU473273 | GU473287 | GU473316 | GU473320 | ------------- |
|  | Edwardsiidae | ***Edwardsia*** | *elegans* | AMNH | EU190726 | EU190770 | EU190857 | EU190815 | GU473338 |
|  |  | ***Edwardsia*** | *japonica* | KUNHM | GU473274 | GU473288 | GU473304 | GU473321 | GU473359 |
|  |  | ***Edwardsia*** | *timida* | KUNHM | GU473281 | GU473299 | GU473315 | ------------- | ------------- |
|  |  | *Edwardsianthus* | *gilbertensis* | AMNH | EU190728 | EU190772 | EU190859 | EU190817 | ------------- |
|  |  | *Metedwardsia* | *akkeshi* | KUNHM | GU473276 | GU473294 | GU473310 | GU473326 | GU473343 |
|  |  | *Nematostella* | *vectensis* | AMNH | EU190750 | AY169370 | AF254382 | EU190838 | FJ489501 |
|  | Halcampidae | *Cactosoma* | sp. nov*.* | AMNH | GU473279 | GU473297 | GU473313 | GU473329 | GU473346 |
|  | Halcampoididae | ***Halcampoides*** | *purpurea* | AMNH | EU190735 | EU190780 | AF254380 | EU190824 | ------------- |
|  | Haloclavidae | ***Haloclava*** | *producta* | AMNH | EU190734 | EU190779 | AF254370 | EU190823 | GU473340 |
| Acontiaria | Aiptasiidae | ***Aiptasia*** | *mutabilis* | KUNHM | ------------- | FJ489418 | FJ489438 | FJ489469 | FJ489505 |
|  |  | ***Aiptasia*** | *pulchella* | KUNHM | EU190715 | EU190757 | EU190846 | EU190803 | FJ489477 |
|  |  | *Bartholomea* | *annulata* | KUNHM | EU190721 | EU190763 | EU190851 | EU190809 | FJ489483 |
|  | Antipodactidae | ***Antipodactis*** | *awii* | AMNH | GU473271 | GU473286 | GU473303 | GU473319 | GU473337 |
|  | Bathyphellidae | ***Bathyphellia*** | *australis* | KUNHM | FJ489402 | FJ489422 | EF589063 | EF589086 | FJ489482 |
|  | Diadumenidae | ***Diadumene*** | ***cincta*** | KUNHM | EU190725 | EU190769 | EU190856 | EU190814 | FJ489490 |
|  | Haliplanellidae | ***Haliplanella* (*)** | ***lineata*** | KUNHM | EU190730 | EU190774 | EU190860 | EU190819 | FJ489506 |
|  | Hormathiidae | *Actinauge* | *richardi* | KUNHM | EU190719 | EU190761 | EU190850 | EU190807 | FJ489480 |
|  |  | *Amphianthus* | sp. | USMN | FJ489413 | FJ489432 | FJ489450 | FJ489467 | FJ489502 |
|  |  | *Calliactis* | *parasitica* | KUNHM | EU190711 | EU190752 | EU190842 | EU190799 | FJ489475 |
|  |  | ***Hormathia*** | *armata* | BEIM | EU190731 | EU190775 | EU190861 | FJ489460 | FJ489491 |
|  | Kadosactidae | ***Kadosactis*** | *antarctica* | BEIM | FJ489410 | EU190782 | EU190865 | EU190825 | FJ489504 |
|  | Metrididae | ***Metridium*** | ***senile*** | KUNHM | EU190740 | EU190786 | AF052889 | EU190829 | FJ489494 |
|  | Nemathidae | ***Nemanthus*** | ***nitidus*** | KUNHM | EU190741 | EU190787 | EU190868 | EU190830 | FJ489495 |
|  | Sagartiidae | *Cereus* | *pedunculatus* | KUNHM | EU190724 | EU190767 | EU190855 | EU190813 | FJ489471 |
|  |  | *Phellia* | *gausapata* | ZSM | EU190744 | EU190790 | EU190870 | EU190833 | FJ489473 |
|  |  | ***Sagartia*** | *troglodytes* | KUNHM | EU190746 | EU190792 | EU190872 | EU190834 | FJ489499 |
|  |  | *Sagartiogeton* | *erythraios* | USNM | ------------- | GU473289 | GU473305 | GU473330 | GU473339 |
|  |  | *Sagartiogeton* | *laceratus* | KUNHM | EU190748 | EU190794 | EU190874 | EU190836 | FJ489500 |
|  |  | *Verrillactis* | *paguri* | KUNHM | FJ489414 | FJ489433 | FJ489440 | FJ489468 | FJ489503 |
| Boloceroidaria | Boloceroididae | ***Boloceroides*** | ***mcmurrichi*** | KUNHM | GU473270 | EU190764 | EU190852 | EU190810 | ------------- |
| Endomyaria | Actiniidae | ***Actinia*** | *fragacea* | CAS | EU190714 | EU190756 | EU190845 | EU190802 | GU473334 |
|  |  | *Anemonia* | *viridis* | CAS | EU190718 | EU190760 | EU190849 | EU190806 | GU473335 |
|  |  | *Anthopleura* | *elegantissima* | KUNHM | EU190713 | EU190755 | EU190844 | EU190801 | GU473333 |
|  |  | *Bunodactis* | *verrucosa* | KUNHM | EU190723 | EU190766 | EU190854 | EU190812 | FJ489484 |
|  |  | *Bunodosoma* | *grandis* | KUNHM | EU190722 | EU190765 | EU190853 | EU190811 | GU473336 |
|  |  | *Epiactis* | *lisbethae* | KUNHM | EU190727 | EU190771 | EU190858 | EU190816 | GU473360 |
|  |  | *Isosicyonis* | *striata* | BEIM | EU190736 | EU190781 | EU190864 | FJ489463 | FJ489493 |
|  |  | *Isotealia* | sp. nov. | FMNH | ------------- | GU473290 | GU473306 | GU473322 | GU473354 |
|  |  | *Macrodactyla* | *doreenensis* | KUNHM | EU190739 | EU190785 | EU190867 | EU190828 | GU473342 |
|  |  | *Urticina* | *coriacea* | KUNHM | GU473282 | EU190797 | EU190877 | EU190840 | GU473351 |
|  | Actinodendridae | *Actinostephanus* | *haeckeli* | KUNHM | ------------- | EU190762 | ------------- | EU190808 | GU473353 |
|  | Aliciidae | *Triactis* | *producta* | KUNHM | EU490525 | ------------- | EU190876 | EU190839 | GU473350 |
|  | Liponematidae | ***Lipomena*** | *brevicornis* | USNM | EU190738 | EU190784 | EU190866 | EU190827 | GU473341 |
|  | Phymanthidiae | ***Phymanthus*** | ***loligo*** | KUNHM | EU190745 | EU190791 | EU190871 | ------------- | GU473345 |
|  | Preactiidae | *Dactylanthus* | *antarcticus* | KUNHM | GU473272 | AY345877 | AF052896 | AY345873 | GU473358 |
|  | Stichodactlyidae | *Heteractis* | *magnifica* | KUNHM | EU190732 | EU190777 | EU190862 | EU190821 | GU473361 |
|  |  | ***Stichodactyla*** | *gigantea* | KUNHM | EU190747 | EU190793 | EU190873 | EU190835 | GU473347 |
| Mesomyaria | Actinoscyphiidae | ***Actinoscyphia*** | *plebeia* | BEIM | EU190712 | EU190754 | FJ489437 | EU190800 | FJ489476 |
|  |  | *Alvinactis* | *chessi* | USNM | GU473278 | GU473296 | GU473312 | GU473328 | GU473352 |
|  |  | *Cyananthea* | *hourdezi* | USNM | GU473275 | GU473293 | GU473309 | GU473325 | GU473364 |
|  | Actinostolidae | ***Actinostola*** | *crassicornis* | USNM | ------------- | EU190753 | EU190843 | EU272904 | GU473332 |
|  |  | ***Actinostola*** | *chilensis* | ZMS | ------------- | GU473285 | GU473302 | ------------- | GU473357 |
|  |  | *Antholoba* | *achates* | ZMS | GU473269 | GU473284 | GU473301 | GU473318 | GU473356 |
|  |  | *Anthosactis* | *janmayeni* | AMNH | ------------- | GU473292 | GU473308 | GU473324 | GU473363 |
|  |  | *Anthosactis* | *pearseae* | CAS | EU190751 | EU190798 | EU190878 | EU190841 | GU473365 |
|  |  | *Hormosoma* | *scotti* | BEIM | EU190733 | EU190778 | EU190863 | EU190822 | GU473366 |
|  |  | *Paranthus* | *niveus* | ZMS | GU473277 | GU473295 | GU473311 | GU473327 | GU473344 |
|  |  | *Stomphia* | *didemon* | KUNHM | EU190749 | EU190795 | EU190875 | EU190837 | GU473348 |
|  |  | *Stomphia* | *selaginella* | AMNH | GU473280 | GU473298 | GU473314 | GU473331 | GU473349 |
|  | Isanthidae | ***Isanthus*** | ***capensis*** | AMNH | ------------- | GU473291 | GU473307 | GU473323 | GU473362 |
|  |  | *Paraisanthus* | *fabiani* | ZMS | ------------- | GU473283 | GU473300 | GU473317 | GU473355 |
| Zoanthidea |  | *Savalia* | *savaglia* | N/A | AY995905 | DQ825686 | HM044299 | HM044298 | DQ825686 |

Generic names in bold indicate type genera; binomens in bold indicate type species of type genera. Samples are organized alphabetically within their current higher taxon. New sequences from this study underlined. (*) As *Diadumene lineata* in Fautin [22], see [43] for a discussion of the nomenclature and taxonomy of this genus.
